# Does social capital interact with economic hardships in influencing older adults’ health? A study from China

**DOI:** 10.1186/s12939-021-01542-y

**Published:** 2021-09-15

**Authors:** Lijuan Gu, Yang Cheng, David R. Phillips, Mark Rosenberg, Linsheng Yang, Li Wang, Hairong Li

**Affiliations:** 1 Key Laboratory of Land Surface Pattern and Simulation, Institute of Geographic Sciences and Natural Resources Research, Chinese Academy of Sciences, 11 A Datun Road, Beijing, 100101 P.R. China; 2 Faculty of Geographical Science, Beijing Normal University, Beijing, 100875 China; 3 Department of Sociology and Social Policy, Lingnan University, Hong Kong, 999077 China; 4 Department of Geography and Planning, Queen’s University, Kingston, ON K7L3N6 Canada; 5 College of Resources and Environment, University of Chinese Academy of Sciences, Beijing, 100875 China

**Keywords:** Physical and mental health, Urban and rural, Cohesion, trust, and participation, Material and psychosocial mechanisms, Older people

## Abstract

**Background:**

The importance of social and economic capital as predictors of health is widely documented, yet the complexity of interactions between them and effects on older people’s health is still unclear. Combining the material and psychosocial explanations of health, this study explores the potential interactions between social and economic capital in influencing older adults’ health in urban and rural China.

**Methods:**

Using data from the China Family Panel Survey, physical and mental health in 2018 were regressed on social and economic capital indicators in 2016, controlling for sociodemographic characteristics of 3535 respondents aged 65 and older. Rothman’s synergy index was calculated to investigate potential interaction effects.

**Results:**

Economic hardships were significantly related to both self-reported health and mental health. Neighborhood cohesion and social participation were significantly associated with mental health for all, bonding trust was significantly associated with mental health for urban older people. We found no significant associations between social capital components and self-reported health. There was an interaction effect between low neighborhood cohesion and economic hardships, and between low social participation and economic hardships, creating an increased burden of poor mental health. The interaction effect between low bonding trust and economic hardships on mental health was apparent only among urban older people.

**Conclusions:**

Geographical settings are important factors in the complexity between social and economic capital in affecting older health. Intervention efforts directed towards reducing simultaneously multiple dimensions of deprivation, such as poverty, social exclusion, social isolation, could be helpful in improving older people’s health. In materially deprived places, policies to promote health equity by improving social capital but without eliminating poverty may be less effective.

## Background

There is ample evidence of social capital [1, 2] and economic capital [3–5] as major social determinants of health. Moreover, the complexity between social and economic capital in affecting health has also been concerned [6, 7]. However, studies on social determinants of health have primarily focused on the general population rather than specific age groups. In this respect, understanding the social determinants of older people’s health is imperative in the context of a rapidly aging world, but published research on the influence of social and economic capital on older people’s health is limited.

China has experienced a rapid process of population aging. This has been accompanied by many changes in perspectives on care of older persons and interest is refocusing on traditional care arrangements guaranteed by adult children. However, in the absence of such guaranteed assistance, older people in China increasingly have to rely more on themselves for their care in old age [7]. In such instance, many other factors, such as neighborhood cohesion and social engagement, which are components of social capital, are therefore relevant [8, 9]. It is also recognized that health in older age is significantly influenced by economic status [10]. The urban–rural dual structure in China has been a major social stratification mechanism underpinning large urban–rural disparities in health, access to social support, educational achievement, health behavior and interpersonal connectedness [7, 8], making urban–rural differences in social determinants of older people’s health highly possible. Therefore, an investigation of the role of social and economic capital in affecting older adults’ health stratified by urban and rural China is very relevant.

### Social capital as a multifaceted concept

As one of the “essentially contested concepts” in social sciences research [9], social capital has entered the mainstream of public health discourse since 1996 [1]. Although it lacks a unified definition, the concept of social capital as social relationships between groups of people is frequently emphasized [11]. Moreover, there is a lack of a standardized approach to measure social capital. In public health research, “social cohesion” and “networks” have been generated as two distinct dimensions [9]. In empirical studies, social capital is often further delineated into structural versus cognitive social capital, and bonding versus bridging social capital [1]. Despite the various domains emphasized in different studies, there is a growing consensus that social capital can be empirically measured by trust, social participation, cohesion, social norms and networks [1, 11, 12]

### Social capital, economic capital and their interaction effect on health

A combination of low social and economic capital can potentially cause multiple restrictions and produce double jeopardy to health. First, a lack of both forms might negatively affect health indirectly through material deprivation. Second, a lack of both might jeopardize health indirectly through social mechanisms [13]. These could be caused by, for example, lessened inclination to participate in social activities and reduced benefits from them. Third, a lack of both might affect health directly through psychosocial pathways as both financial difficulties and social isolation are closely related to psychological stress and adverse health outcomes [13]. Another possible reason for the potential double jeopardy on health lies in the reciprocal relationships between low social capital and economic hardships [14, 15].

Completeness in describing cause and effect relationships warrants evaluation of interactions [16]. In epidemiology, “interaction” refers to the extent to which the effect of joint exposure on health outcomes differs from the independent effect of single exposure. Very few studies have empirically examined the potential interactions of social and economic capital on health. One study based on a sample of 1605 participants in two Chinese cities found an interaction between low social capital and poverty in predicting poor health [5]. A Swedish study observed significant interactions between economic hardships and various types of social capital on self-reported health, psychological distress and musculoskeletal disorders [13]. These two studies focus on the general population and studies empirically examining the potential interactions of social and economic capital on older people’s health are very rare.

### Social capital, economic capital and older people’s health in urban and rural China

China’s unprecedented economic growth in the past four decades has been accompanied by gradual social changes [17, 18]. Massive labor migration, rapid urbanization and low birth rate have placed considerable strains on China’s traditionally dominant social capital based on ancestral relationships and extended family networks. A modern social capital system based on cooperation and extensive trust is gradually emerging [5]. Instead of family and work units, the neighborhood is playing an increasingly important role in providing social and welfare support. Unlike the Western world, where neighborhood is more of an autonomous and informal association, neighborhood in China is designated as the grassroots unit of administration [19]. Residents of neighborhoods normally share little in common and sense of community is still being developed [20]. Moreover, unlike many other contexts, where grassroots non-government organizations (NGOs) have few government ties, NGOs in China hinges heavily on the mutual needs of both the associations and the state [21].

China’s structured urban–rural distinction has created the macrolevel environment with which older people have had continual transactions. With rapid socioeconomic developments, the traditional reliance on in-group connections has decreased, and urban dwellers are gradually becoming accustomed to handling affairs according to contracts, rules and regulations [22]. By contrast, because of its relatively slow flow of population, there are well-connected social networks in rural China. However, the policies and programs for promoting age-friendly communities in China are very much city-centric, leaving millions of “left-behind”, low-income older adults in rural areas [23]. Moreover, the huge urban–rural inequalities in earnings, economic support and health resources have created a considerable urban–rural gap in health outcomes [7]. Addressing the socioeconomic explanations of this disparity in health is vital if China is to realize “Active Aging”.

Major differences in socioeconomic conditions between urban and rural China warrant separate analyses [17], and especially their effects on older people. Previous studies linking social capital to health taking residence into account have mainly concentrated on the general population. There are very few studies of the associations between social capital and older adult’s health by residence in China. Two studies reported significant associations between bonding trust, associational membership and health only in the urban areas [8, 24]. However, one study found no urban–rural heterogeneity in their associations [25]. Clearly, far more evidence is needed to understand the urban/rural differences in the relationship between social capital and health. Moreover, an exploration of the interaction effects of social and economic capital on older people’s health is imperative.

### The present study

In accounting for health inequalities, traditional materialist approaches stress poverty as disadvantageous to health, and neo-materialist explanations stress the key role of material resources [26]. In comparison, psychosocial explanations tend to highlight the importance of socioeconomic position and psychosocial stress [27]. Neither of these explanations can fully explain health inequality and they are not mutually exclusive [24]. Given the difficulties of disentangling these two explanations, the complex interactions between social and economic capital in influencing older adults’ health through both material and psychosocial pathways will be considered.

Considering the development of social capital in the Chinese context, we use neighborhood cohesion, trust, social participation and perception of safety as social capital components. The purpose of this study is to obtain a thorough understanding of the interaction between social and economic capital and their effects on older people’s physical and mental health in both urban and rural China. To achieve this purpose, we first analyze the urban/rural distribution of social and economic capital. We then examine the associations between social and economic capital. We further investigate the potential impacts of social and economic capital on health. Finally, we explore whether there are interaction effects between social and economic capital. We hypothesize that:

1. There are urban/rural differences in levels of social capital. We expect (a) lower levels of neighborhood cohesion and trust in urban China and (b) lower levels of social participation in rural China.
2. There is a close correlation between social participation and economic capital.
3. Indicators of social capital are closely associated with health and there are urban/rural differences in these associations.
4. There are significant interactions between social and economic capital in affecting health and there are urban/rural differences in these interactions.

## Methods

### Data source

The present study investigated data from Wave 4 (2016) and Wave 5 (2018) of the China Family Panel Studies (CFPS) (http://www.isss.pku.edu.cn). The CFPS is a nationally representative longitudinal survey conducted every 2 years on the evolution of the Chinese society, economy, population, and health. Covering 25 of the 31 mainland provinces and representing 95% of the Chinese population, CFPS offers the most comprehensive and high-quality data of contemporary China [28]. There were 3703 community-dwelling older adults aged from 65 to 95 years who had participated in both wave 4 and wave 5. For ease of the urban–rural comparison, 168 samples who had changed their place of urban/rural residence were excluded. Finally, 1712 urban and 1823 rural older adults were included.

### Measures

#### Heath outcomes

Self-reported health was used to capture overall health and the 8-item version of the Center of Epidemiologic Studies-Depression scales (CES-D-8) was used to measure mental health. Being a reliable measure that predicts the risk of mortality in older people [29], SRH is dichotomized into “healthy” (0) combining very good, good and fair and “unhealthy” (1) combining poor and very poor. CES-D-8 is a reliable measure of depression symptoms in community-dwelling older people [30]. In CFPS, CES-D was scaled from 0 to 24 with higher values indicating worse mental health. Referencing clinical practice [31], 9 was used as the cut-off to identify clinically significant depression symptoms (Table 3).

#### Social capital

Four components in terms of 10 indicators concerning dimensions of neighborhood cohesion, perception of trust, social participation and perception of neighborhood safety were initially selected (Table 1). Factor analysis was conducted to verify our categorization and to extract independent indicators. After several rounds of analyses, four factors, namely neighborhood cohesion, bridging trust, bonding trust, and social participation, were extracted (perception of trust in people meet for the first time and perception of safety was excluded to avoid ambiguity because of their modest factor loadings on all factors) (Table 2). The values of factors were standardized and dichotomized into binary variables using zero as the switch point (Table 3).

**Table 1:** Social capital components and indicators

Components	Indicators
Neighborhood cohesion	How is the relationship between neighbors in your community?
	When you need any help from neighbors, will someone lend you a hand?
	Are you emotionally attached to your neighborhood?
Perception of trust	How much do you trust your parents?
	How much do you trust your neighbors?
	How much do you trust people you meet for the first time?
	How much do you trust your doctors?
	How much do you trust the local government officials?
Social participation	How many cultural/ political/ civic/ religious/ any other social groups or organizations have you participated in?
Perception of safety	How do you rate the public safety in your neighborhood?

**Table 2:** Factor loadings of social capital indicators

Indicators	Fac1	Fac2	Fac3	Fac4	Communality
	NC	BRT	BOT	SP	
How is the relationship between neighbors in your community?	0.735	0.098	0.098	0.033	0.560
When you need any help from neighbors, will someone lend you a hand?	0.753	0.010	0.083	0.017	0.574
Are you emotionally attached to your community?	0.756	0.136	-0.018	-0.030	0.592
How much do you trust your doctors?	0.059	0.829	0.125	-0.053	0.709
How much do you trust your local government officials?	0.105	0.860	0.010	0.063	0.754
How much do you trust your parents?	0.015	0.036	0.942	0.001	0.889
How much do you trust your neighbors?	0.322	0.432	0.501	0.027	0.542
How many social groups or organizations have you participated in?	0.014	0.010	0.011	0.997	0.995

1. KMO: 0.71; Sig. of Bartlett’s Test: 0.000; Total variance explained: 70.201%

2. NC: neighborhood cohesion; BRT: bridging trust; BOT: bonding trust; SP: social participation

3. Initial Eigen value: 1.798 (NC), 1.643 (BRT), 1.171 (BOT), and 1.004 (SP)

**Table 3:** Descriptive statistics of the total, urban and rural older residents

		Total (*N* = 3535)	Urban (*N* = 1712)	Rural (*N* = 1823)
SRH in 2018 (bad, %)^a^		30.4	25.7	34.9
SRH in 2016 (bad, %)^b^		28.6	24	30.2
CESD in 2018 (≥ 9, %)^c^		25.3	20	32.9
CESD in 2016 (≥ 9, %)^d^		22.7	17.5	27.6
Age (M/SD)^e^		72.89/5.087	73.3/5.421	72.52/4.723
Gender (male, %)^f^		51.7	49.8	53.5
Education(%)^g^				
No formal education		48.8	38.6	58.3
Primary school		28.6	27.9	29.2
Middle school		14.5	19.6	9.7
High school		5.5	8.9	2.2
College or higher		2.7	5	0.5
Marital status (%)^h^				
Married/cohabit		73.8	75.2	72.5
Divorced		1.1	1.1	1.1
Widowed		24.2	23.3	25.1
Single		0.9	0.5	1.3
Smoking in the past month (%)^i^				
Yes		26.3	22.7	29.7
Drinking alcohol for at least 3 times a week (%)				
Yes		17.7	17.6	17.7
Family size (%)^j^				
1	8.7		8.3	9.1
2	36.4		38.8	34.2
3	11.1		12.4	9.8
4	10.6		10.2	11
5	14.7		15.7	13.7
6 +	18.5		14.5	22.3
Economic hardships (yes, %)^k^	15.5		7.7	22.8
Neighborhood cohesion (low, %)^l^	39.1		42	36.4
Bridging trust (low, %)	40.5		42.1	38.9
Bonding trust (low, %)	38		37.9	38.2
Social participation (low, %)^m^	74		64.7	82.8

Statistics in this table were calculated based on complete data

^a^χ^2^(1,*N* = 1) = 35.93, *P* < .001; ^b^χ^2^(1,*N* = 1) = 34.04, *P* < .001; ^c^χ^2^(1,*N* = 1) = 48.12, *P* < .001; ^d^χ^2^(1,*N* = 1) = 48.09, *P* < .001; ^e^*t*(3533) = 35.93, *P* < .001; ^f^χ^2^(1,*N* = 1) = 4.88, *P* = .029; ^g^χ^2^(1,*N* = 4) = 269.829, *P* < .001; ^h^χ^2^(1,*N* = 3) = 9.06, *P* = .028; ^i^χ^2^(1,*N* = 1) = 21.75, *P* < .001; ^j^χ^2^(1,*N* = 5) = 43.41, *P* < .001; ^k^χ^2^(1,*N* = 1) = 152.52, *P* < .001; ^l^χ^2^(1,*N* = 1) = 10.963, *P* = .001; ^m^χ^2^(1,*N* = 1) = 139.978, *P* < .001

#### Economic hardships

Three indicators of financial difficulties were used: (i) low disposable household income per capita (ii) low individual income (iii) inability to meet expenses in the past 12 months. We calculated disposable household income per capita by dividing the total disposable household income by household size. Following extant practice [13, 32], the lowest quintile was defined as low. Survey questions on annual individual income were used to measure individual income. Similarly, the lowest quintile was defined as low. The CFPS survey inquired of respondents’ total family income and expenditure in the past 12 months. Participants with the family’s total expenditure being higher than total income were redeemed to be unable to meet expenses. Reliability analysis indicated a low level of internal reliability (Cronbach’s α value = 0.374). However, in defining economic hardships, it was felt more reasonable to combine different measures of economic hardships to capture various dimensions of its multifaceted construct [5, 15]. Referring to published research [13], we constructed a composite index of economic hardships by summing the values of these three indicators with 0 and 1 indicating no economic hardships, and 2 and 3 indicating economic hardships.

#### Covariates

Gender, age, educational attainment, marital status, the family size, smoking behavior and drinking behavior were used as control variables. See Table 3 for details.

### Analytical strategy

To understand the complexity between economic and social capital and in influencing older people’s health, we first examined the correlation between economic hardships and low social capital. Second, we investigated the impact of economic hardships and low social capital on health. Finally, we explored the potential interaction effects of low social capital and economic hardships. To minimize the potentially reverse causal relationship, we analyzed the impact of social and economic capital at wave 4 on the health outcomes at wave 5, controlling for health at wave 4 and other covariates. There were two reasons for controlling for previous health status: first, one person’s health status at wave i will usually be heavily dependent on his/her health status at wave i-1. Second, there will be unobserved time-invariant factors, such as personality and biological traits, which might influence a person’s health status on any occasion. Controlling for previous health status can allow for this kind of autocorrelation caused by unobserved heterogeneity. By regressing individual level health outcomes in 2018 on individual level social and economic capital indicators in 2016, our study was a cohort study. Therefore, stronger evidence of causal relationship was provided. Moreover, the potential “ecological fallacy” trap in using aggregated data [33], which would be caused by ascribing group-level associations to the individual, was avoided.

Cross-tabulations were used to see if significant associations existed between social capital and economic hardships. Multivariate logistic regression analyses with lagged effects were used to estimate the impacts of social and economic capital on health. In model 1 s, with the adjustment of previous health status, social capital components and economic hardships were separately included. In model 2 s, with the adjustment of previous health and covariates, social capital components and economic hardships were separately included. In model 3 s, with the adjustment of previous health and covariates, social capital components and economic hardships were simultaneously included.

We applied the synergy index (SI) to quantify interaction. To calculate the SI, we first used multivariate logistic models with lagged effects to estimate adjusted odds ratios (OR) with joint exposure, single exposure and no exposure. We then applied Kalilani and Atashili’s formulas to calculate risk ratio (RR) [34]. We finally calculate the SI [16]. The equations are:

$$\begin{array}{*{20}l} {RR_{10} = \frac{{OR_{10} \left( {1 + O_{00} } \right)}}{{1 + O_{10} }}} \hfill \\ {RR_{01} = \frac{{OR_{01} \left( {1 + O_{00} } \right)}}{{1 + O_{01} }}} \hfill \\ {RR_{11} = \frac{{OR_{11} \left( {1 + O_{00} } \right)}}{{1 + O_{11} }}} \hfill \\ {SI = \frac{{RR_{11} - 1}}{{\left( {RR_{10} - 1} \right) + \left( {RR_{01} - 1} \right)}}} \hfill \\ \end{array}$$

Where $RR_{10}$ denotes the risk ratio to report poor health with exposure to economic hardships, $RR_{01}$ denotes the risk ratio with exposure to low social capital, and $RR_{11}$ denotes the risk ratio with both exposures. $OR$ denotes adjusted odds ratios from logistic regression analysis. $O$ denotes odds. The value of SI would exceed 1 for causes acting together creating a greater joint effect, would equal 1 for causes acting independently, and would be less than 1 for causes jointly create a weaker effect.

Except for gender, age and education, other variables involved had missing data, with proportions ranging from 0.03% for SRH to 7.5% for social participation. The majority were not missing completely at random. To reduce estimation bias, multiple imputation was applied. Given that we had 17 partially observed variables and both continuous and categorical variables were covered, multiple imputation using chained equations (ICE) was used [35]. Considering the proportion of missing data and the efficiency of estimation, we imputed 20 datasets and used Rubin’s rules to combine the estimates and standard errors of these 20 imputations.

## Results

### Descriptive statistics

Table 1 shows descriptive characteristics. Compared to 2016, there was an increasing trend in the proportions reporting poor health in 2018. The proportions of participants reporting poor health were higher in rural areas. Generally, older adults in rural areas reported a higher level of economic hardships (22.8 vs. 7.7%). Urban older people reported a higher proportion of low neighborhood cohesion (42 vs. 36.4%), whereas rural older people reported a higher proportion of low social participation (82.8 vs. 64.7%). There was no significant urban–rural difference in bridging and bonding trust.

### Correlation between low social capital and economic hardships

Table 4 lists the cross-tabulation of social capital and economic hardships. Neither low neighborhood cohesion nor low trust were significantly correlated with economic hardships. Low social participation was significantly correlated with economic hardships.

**Table 4:** Cross-tabulation of social capital components and economic hardships for total, urban and rural respondents (%)

	Economic hardships		χ2	df	*P* value
	No	Yes			
**Total**					
Low neighborhood cohesion					
No	60.90	60.80	0.00	1	1.000
Yes	39.10	39.20			
Low bridging trust					
No	59.10	62.20	1.78	1	0.197
Yes	40.90	37.80			
Low bonding trust					
No	61.90	62.20	0.02	1	0.920
Yes	38.10	37.80			
Low social participation					
No	28.30	13.30	49.48	1	< 0.001
Yes	71.70	86.70			
**Urban**					
Low neighborhood cohesion					
No	57.70	61.30	0.597	1	0.499
Yes	42.30	38.70			
Low bridging trust					
No	57.20	66.40	3.786	1	0.054
Yes	42.80	33.60			
Low bonding trust					
No	62.10	63.00	0.042	1	0.922
Yes	37.90	37.00			
Low social participation					
No	36.50	21.00	11.55	1	0.001
Yes	63.50	79.00			
**Rural**					
Low neighborhood cohesion					
No	64.50	60.70	1.84	1	0.182
Yes	35.50	39.30			
Low bridging trust					
No	61.10	60.90	0.004	1	0.952
Yes	38.90	39.10			
Low bonding trust					
No	61.70	62.00	0.009	1	0.952
Yes	38.30	38.00			
Low social participation					
No	19.00	10.80	13.88	1	< 0.000
Yes	81.00	89.20			

Statistics in this table were calculated based on complete data

### Association between low social capital, economic hardships and health

Table 5 shows the logistic estimations of reporting poor health in the urban areas. Both economic hardships and low social participation were significantly and separately associated with poor SRH. This significant association persist in model 2 s. However, in model 3 s, only the relationship between economic hardships and poor SRH persisted. In comparison, the significant associations between economic hardships, low neighborhood cohesion, low bonding trust, low social participation and poor mental health were robust in all models. Table 6 shows the logistic estimations of the rural respondents. Economic hardships, low bridging trust and low social participation were significantly associated with poor SRH in model 1 s. However, in model 2 s and model 3 s, they were no longer significant. Comparatively, the significant associations between economic hardships, low neighborhood cohesion, low social participation and poor mental health were robust through all models.

**Table 5:** Odds ratios for reporting poor health in relation to economic hardships and low social capital in urban areas

	Poor SRH			Poor mental health		
	OR (95% CI)			OR (95% CI)		
	Model 1	Model 2	Model 3	Model 1	Model 2	Model 3
Urban (*N* = 1712)						
Economic hardships						
Yes	1.74 (1.2,2.52)^b^	1.74 (1.18,2.57)^b^	1.73 (1.17,2.55)^b^	1.9 (1.29,2.82)^c^	1.69 (1.11,2.57)^a^	1.71 (1.12,2.62)^a^
Neighborhood cohesion						
Low	1.25 (0.99,1.56)	1.25 (0.99,1.58)	1.24 (0.99,1.56)	1.39 (1.09,1.78)^b^	1.53 (1.19,1.99)^c^	1.52 (1.17,1.97)^b^
Bridging trust						
Low	1.05 (0.83,1.31)	1.04 (0.82,1.32)	1.05 (0.83,1.33)	1.22 (0.95,1.57)	1.25 (0.96,1.63)	1.26 (0.96,1.64)
Bonding trust						
Low	0.98 (0.77,1.24)	0.97 (0.77,1.24)	0.98 (0.77,1.24)	1.36 (1.05,1.77)^a^	1.31 (1,1.72)^a^	1.33 (1.01,1.76)^a^
Social participation						
Low	1.4 (1.09,1.8)^b^	1.32 (1.01,1.73)^a^	1.29 (0.98,1.69)	1.85 (1.4,2.44)^c^	1.45 (1.07,1.96)^a^	1.39 (1.02,1.89)^a^

Model 1: Adjusted for previous health status. The four social capital components and economic hardships were separately included

Model 2: Adjusted for previous health status, age, gender, educational attainment, marital status, smoking behavior, drinking behavior and family size. The four social capital components and economic hardships were separately included

Model 3: Adjusted for previous health status, age, gender, educational attainment, marital status, smoking behavior, drinking behavior and family size. The four social capital components and economic hardships were simultaneously included ^a^p<0.05, ^b^p<0.01, ^c^p<0.001

**Table 6:** Odds ratios for reporting poor health in relation to economic hardships and low social capital in rural areas

	Poor SRH			Poor mental health		
	OR (95% CI)			OR (95% CI)		
	Model 1	Model 2	Model 3	Model 1	Model 2	Model 3
Rural (*N* = 1823)						
Economic hardships						
Yes	1.65 (1.32,2.06)^c^	1.51 (1.2,1.9)^c^	1.49 (1.18,1.88)^c^	1.58 (1.25,1.99)^c^	1.48 (1.16,1.89)^b^	1.42 (1.11,1.81)^b^
Neighborhood cohesion						
Low	1.16 (0.94,1.43)	1.18 (0.95,1.47)	1.16 (0.93,1.44)	1.61 (1.31,1.98)^c^	1.63 (1.31,2.02)^c^	1.61 (1.29,2.00)^c^
Bridging trust						
Low	0.79 (0.64,1.08)	0.81 (0.65,1.23)	0.8 (0.65,1.29)	1.09 (0.87,1.36)	1.11 (0.88,1.40)	1.1 (0.87,1.39)
Bonding trust						
Low	1.08 (0.87,1.35)	1.08 (0.86,1.35)	1.09 (0.87,1.36)	1.22 (0.98,1.51)	1.21 (0.97,1.51)	1.16 (0.93,1.46)
Social participation						
Low	1.45 (1.09,1.93)^a^	1.23 (0.92,1.67)	1.2 (0.89,1.62)	2.07 (1.50,2.85)^c^	1.84 (1.31,2.58)^c^	1.83 (1.30,2.57)^c^

Model 1: Adjusted for previous health status. The four social capital components and economic hardships were separately included

Model 2: Adjusted for previous health status, age, gender, educational attainment, marital status, smoking behavior, drinking behavior and family size. The four social capital components and economic hardships were separately included

Model 3: Adjusted for previous health status, age, gender, educational attainment, marital status, smoking behavior, drinking behavior and family size. The four social capital components and economic hardships were simultaneously included  ^a^p<0.05, ^b^p<0.01, ^c^p<0.001

### The interaction effects of low social capital and economic hardships on health

**Fig. 1:**
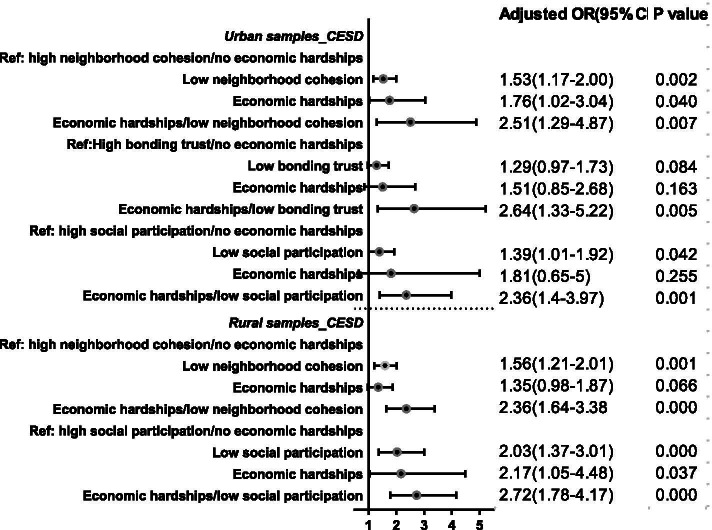
Odds ratios, 95% confidence intervals, and *p*-values for reporting poor mental health associated with social capital components according to levels of economic hardships for interactions. Note: Only the interactions of economic hardships and social capital components exhibiting independently significant impacts on health were included. Model adjusted for mental health status in 2016, gender, age, educational attainment, marital status, smoking behavior, drinking behavior, and family size

Figure 1 maps the adjusted ORs with combinations of low social capital components and economic hardships. In the urban areas, if older adults were exposed to either low neighborhood cohesion or economic hardships, the ORs were 1.53 and 1.76; if exposed to both, the OR increased to 2.51. If exposed to either low bonding trust or economic hardships, the ORs were 1.19 and 1.51; if exposed to both, the OR was 2.64. If exposed to either low social participation or economic hardships, the ORs were 1.39 and 1.81; if exposed to both, the OR was 2.36. In the rural areas, if exposed to either low neighborhood cohesion or economic hardships, the ORs were 1.56 and 1.35; if exposed to both, this value was 2.36. If exposed to either low social participation or economic hardships, the ORs were 2.03 and 2.17; if exposed to both, this value was 2.72.

**Fig. 2:**
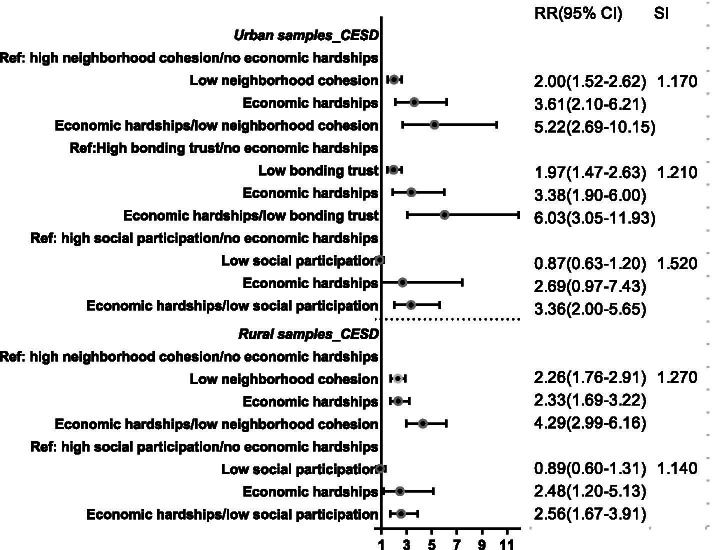
Risk ratios, 95% confidence intervals, and Synergy Index for reporting poor mental health associated with social capital components according to levels of economic hardships for interactions. Note: Only the interactions of economic hardships and social capital components exhibiting independently significant impacts on health were included. Model adjusted for mental health status in 2016, gender, age, educational attainment, marital status, smoking behavior, drinking behavior, and family size

Figure 2 depicts the RRs and synergy index (SI). In the urban areas, the SI between low neighborhood cohesion and economic hardships was 1.17, the SI between low bonding trust and economic hardships was1.21, and the SI between low social participation and economic hardships was 1.52. In the rural areas, the SI between low neighborhood cohesion and economic hardships was 1.27, and the SI between low social participation and economic hardships was 1.14.

## Discussion

Our study has revealed interesting interactions between indicators of social capital and economic hardships in influencing older people’s health, and important differences in the level of social capital and the interactions between social capital and economic hardships in affecting health between urban and rural older people. Specifically, there was a higher level of neighborhood cohesion among rural older people and a higher level of social participation among the urban ones. Low social participation was significantly correlated with economic hardships. Economic hardships were positively and independently associated with poor SRH and mental health, and low neighborhood cohesion and low social participation were positively and independently associated with poor mental health for all. In comparison, low bonding trust was positively associated with poor mental health only in urban areas. There was an interaction effect between low neighborhood cohesion and economic hardships, and between low social participation and economic hardships, creating an increased burden of poor mental health. The interaction effect between low bonding trust and economic hardships on mental health was only among urban older people.

Our findings of urban/rural differences in the level of social capital are generally consistent with Hypothesis 1. With the recent rapid economic development and social transition, traditional in-group connections appear to decrease and urban people normally handle affairs according to rules and contracts [22]. In comparison, in rural areas, social relationships are dominated by the tight connections between traditional kinship networks [8]. Therefore, the level of neighborhood cohesion in rural China is higher than in urban areas. We used the number of cultural/ political/ civic/ religious/ any other social organizations in which older people had participated to measure social participation which, in China, is mainly initiated by the government and led by Communist Party members [20]. However, the expansion of Communist Party membership in rural China has been hindered because of lower level of education and clan effects [8]. Therefore, as previously indicated [36], there are broader opportunities for social participation in the urban areas. Interestingly, we found no significant urban–rural differences in the level of trust, which is unexpected and different from other studies which have tended to indicate a higher level of bonding trust among rural people [8, 37]. A possible explanation is that previous studies used data from 2005, and we used data from 2016. Urbanization has dramatically increased recently and especially in the last decade and the disintegration of the big family has challenged the traditional older age care model. The sometimes called “left-behind” rural older people are very likely to develop an empty nest syndrome after their children have left or migrated and may feel socially isolated [23]. Eventually, their level of trust decreases and the urban–rural differences in trust become less marked.

This study shows that low social participation was significantly correlated with economic hardships. This finding is understandable in that, unlike most of the Western world, where organizational membership is normally voluntary, participation in social organizations in the Chinese context should be formally registered, and a formal membership always requires relatively high socioeconomic status [21]. Different from previous studies which indicated a close association between social capital and income [5, 38], this study found no significant relationship between cohesion, trust and economic hardships. One possible explanation is in the complexity and variability of these relationships across cultures. Moreover, trust may be the product of previous knowledge and does not require continual investment of resources, and cohesion mainly requires investment of time and does not require continual investment of material resources either [38].

The third main finding is that social capital components had significant but differential associations with mental health in urban and rural China. The association between bonding trust and mental health among only urban older people echoes previous research [8]. By contrast, limited education might be a barrier for rural older people to fully utilize their social networks, thereby restricted them from profiting directly from bonding trust [8]. In line with previous studies [39, 40], this current study found an association between low neighborhood cohesion and poor mental health in both urban and rural areas. This finding is supported by previous arguments that a cohesive neighborhood could influence health via psychosocial pathways by providing affective support, mutual respect and self-esteem  [41].

We found significant associations between social participation and older adults’ mental health in both the urban and rural areas, which is in accordance with previous research  [36]. By contrast, some previous studies reported this association among only urban older people  [8, 42], and some found no significant association [5, 17]. It is widely argued that participation in social activities is beneficial for mental health  [43, 44]. However, it is also believed that many Chinese people prefer to participate in informal groups, while the majority of organizations need to be formally registered, which causes a lack of association between social participation and health [5]. Clearly, there is a need to analyze this association further in future research. Our findings indicated no significant associations between social capital and SRH. Previous studies empirically investigating the relationships between social capital and older adults’ SRH are limited and there is a lack of conclusive associations [1]. One possible reason may be that material resources are more critical to physical health, especially for older people [8]. However, the explanation warrants further in-depth exploration.

 As hypothesized, our fourth main finding reveals an interaction effect between low social capital and economic hardships in both urban and rural areas. This extends previous findings on the synergy effect between social capital and economic capital on the health of the general population [5, 13]. As discussed earlier, low social capital and economic hardships may generate aggravating effects on older people’s health through material, social and psychosocial pathways. Moreover, a lack of social and economic capital can be regarded as multiple forms of marginalization or discrimination, which can create a spiral of cumulative disadvantage  [13, 45], especially for frail and vulnerable older people. The overlapping forms of marginalization or discrimination may magnify any detrimental effects of single factors on health  [45]. Structural conditions are sometimes more powerful than individual-level limitations and, in this case, socioeconomic indicators may not be able to capture all influences affecting health [46, 47]. Given China’s structural urban–rural distinction affecting various aspects of life and resources, the interaction between bonding trust and economic hardships among only urban older people is understandable.

Some limitations to this study should be noted. First, due to the inconsistent survey questions across waves, we only examined the potential impacts of social and economic capital in 2016 on health outcomes in 2018. Although our strategy may help to reduce the possibility of reversal causal inference, we are cautious of any conclusion regarding causality. Second, because of the small proportion of older residents surveyed in each neighborhood, this study was unable to analyze the potential complexity between social and economic capital in affecting health at the contextual level. Future studies covering both individual and contextual measures of social and economic capital are needed to further understand their potential interactions and influences on older people’s health. Finally, we only included community-dwelling older adults and stratified them only by urban/rural residence. Therefore, potentially more frail older adults living in institutions were not included. Moreover, there were approximately 50 million left-behind older people in rural China by 2015, and whether someone is left behind or not left behind is a growing determinant in older persons’ health interventions [2]. Future research should therefore if possible cover a wider range of the aging population and conduct group-targeted analyses.

## Conclusions

Evidence on the complex associations between social and economic capital in influencing older pepople’s health is limited, although understanding social determinants of health is urgent and imperative in the context of rapid demographic aging. To our knowledge, this study is one of the very first to investigate the complex interactions between social and economic capital in affecting older people’s health in both urban and rural China. Our data support the contention that there are siginifcant differences in social and economic capital between urban and rural China. Economic hardships, neighborhood cohesion and social participation were independently associated with mental health for urban and rural older people and bonding trust was associated with the mental health of urban older people. Further analysis indicates an aggravating effect between low neighborhood cohesion and economic hardships, between low bonding trust and economic hardships, and between low social participation and economic hardships, creating an increased burden of poor mental health. Our results add important new evidence of the urban/rural differences in the social determinants of older people’s health, extend our understanding of the relevance between social and economic capital in influencing health. These could inform and lead to more effective interventions to improve older people’s health and to reduce health disparities.

Intervention efforts directed at simultaneously reducing multiple dimensions of deprivation, such as poverty, social exclusion and social isolation, should be helpful in effectively improving older people’s health. In materially deprived regions, practices aiming at promoting health equity by improving social capital without eliminating poverty may be inadequate. Our findings will also offer insights for other populations with similar socioeconomic conditions and cultural backgrounds.

## Data Availability

The data that support the findings of this study are available from the Institute of Social Science Survey of Peking University but restrictions apply to the availability of these data, which were used under license for the current study, and so are not publicly available. Data are however available from the authors upon reasonable request and with permission of the Institute of Social Science Survey of Peking University.
